# Severe Chronic Suppurative Osteomyelitis Following Dental Implant Placement

**DOI:** 10.1155/crid/6770915

**Published:** 2025-02-24

**Authors:** Marcel da Silva Garrote, Alexandre Augustus Costa Barbosa, Maria de Fátima Batista Medeiros Alves Teixeira, Elismauro Francisco Mendonça, Gilberto Fenelon, Orlando Aguirre Guedes, Carlos Estrela

**Affiliations:** ^1^Department of Oral and Maxillofacial Surgery, School of Dentistry, Pontifical Catholic University of Goiás, Goiânia, Goiás, Brazil; ^2^Department Infectious Diseases, Tropical Disease Hospital, Goiânia, Goiás, Brazil; ^3^Unique Hospital, Goiânia, Goiás, Brazil; ^4^Department of Oral Pathology, School of Dentistry, Federal University of Goiás, Goiânia, Goiás, Brazil; ^5^Neurologic Hospital, Goiânia, Goiás, Brazil; ^6^Department of Oral Biology, School of Dentistry, Evangelical University of Goiás, Anápolis, Goiás, Brazil; ^7^Department of Stomatology, School of Dentistry, Federal University of Goiás, Goiânia, Goiás, Brazil

**Keywords:** dental implant, implant failure, infection, oral surgery, osteomyelitis

## Abstract

Osteomyelitis is an infection caused by bacterial contamination of the bone marrow, cortical surfaces, and the periosteum. The clinical examination of a patient with severe osteomyelitis secondary to dental implants revealed a large facial swelling, suppuration in the perimandibular region, limited mouth opening, and diffuse pain that started after the placement of five dental implants. Two failed implants were removed, a reconstructive titanium plate was placed, and the patient was treated with antibiotics, but the infection did not resolve. The patient's clinical condition became worse, and she sought hospital care. At presentation, she had a fever and was dehydrated. She was hospitalized for 16 days. Her treatment included surgery for the removal of the reconstructive plate, the implants with peri-implantitis, and bone and necrotic tissue, together with administration of systemic drugs. The diagnosis of osteomyelitis was based on clinical, imaging, and histopathological findings, and she was treated with administration of antibiotics (penicillin, amikacin) for 16 days, followed by cephalosporin for 15 days after hospital discharge. Sixteen days later, her clinical condition was normal. Twelve months later, she received new implants and underwent prosthetic rehabilitation. Imaging tests, surgical elimination of bone and necrotic tissue, and histopathological analyses are essential for an accurate diagnosis. In our case, infection control demanded a careful surgical intervention associated with the administration of systemic antibiotics.

## 1. Introduction

Osteomyelitis is an infection of bone marrow spaces, cortical surfaces, periosteum, and the Haversian and Volkmann's canals due to local or blood contamination by bacteria [1]. The early diagnosis and treatment of osteomyelitis are essential to avoid the complex consequences of this infection, such as pain, swelling, purulent collection, amputation, and even death [2]. Unfortunately, its diagnosis and treatment are often delayed [1–3].

Osteomyelitis is commonly associated with trauma (bone fracture), firearm accidents, infections, and spread of localized osteitis [4]. Long bones, such as the mandible, are the most affected because of the characteristic of their vascular supply and reduced bone marrow spaces, which make it difficult to contain exudate and inflammatory infiltrates [5].

Clinically, osteomyelitis is characterized by pain, localized swelling, and inflamed and painful lymph nodes. Sometimes, there is a purulent discharge from the skin or mucosa. This infection may affect men and women at any age but seems to be more frequent among adult men with compromised resistance to systemic diseases [1, 5]. Imaging studies usually show a radiolucent, ill-defined, and irregular “hatchet” appearance, often with radiopaque sequestrums [2, 6]. Maxillofacial osteomyelitis may originate from several types of infections, such as dental, endodontic, or periodontal infections, postsurgical infections, and infections associated with dental implants. Mandibular osteomyelitis usually results from mixed infections [3, 6–10].

In general, osteomyelitis is treated by draining and removing bacterial colonies attached to necrotic bone sequestrums in infected areas and by the administration of antibiotics to prevent dissemination and additional complications [2, 5, 9].

Previous studies [11, 12] found that dental implant failure may be associated with several factors: the patient's underlying medical condition, smoking, bone quality, bone grafting, radiotherapy, parafunctional habits, operator's experience, degree of surgical trauma, bacterial contamination, no administration of preoperative antibiotics, immediate loading, nonsubmerged implants, number of implants supporting a prosthesis, characteristics of the implant surface, and implant design.

Bone infections may occur after the placement of dental implants [2, 3, 6–8]. This report describes an unusual case of severe chronic suppurative osteomyelitis in a 57-year-old woman after the placement of several dental implants.

## 2. Case Report

**Figure 1:**
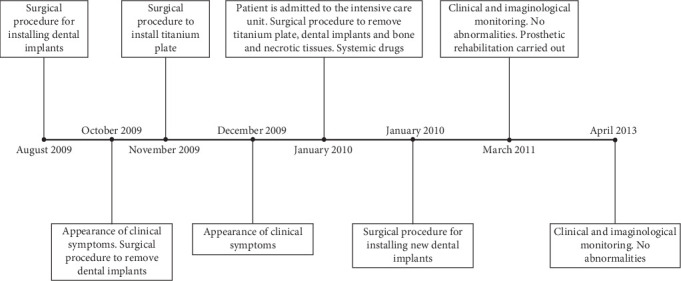
Timeline illustrating the treatment period, including key events and interventions.

**Figure 2:**
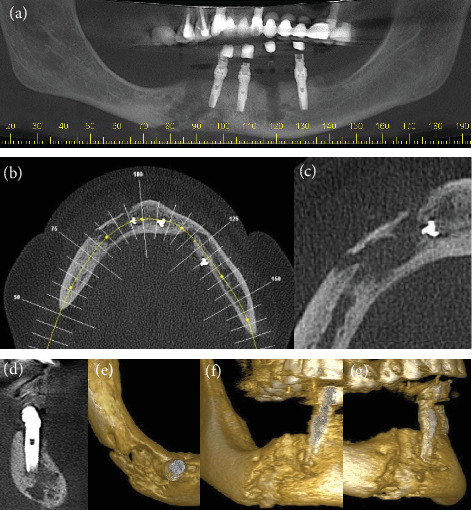
(a–g) CBCT scans show the osteolytic and radiolucent area with cortical destruction of the buccal plate and base of the right quadrant of the mandible. (e–g) 3D reconstruction shows bone destruction.

In January 2010, a 57-yead-old female patient, nonsmoker, with no relevant medical history, was admitted to the Department of Oral and Maxillofacial Surgery of the Neurologic Hospital of Goiânia, Brazil. She presented with a large facial swelling, suppuration in the perimandibular region, limited mouth opening, and diffuse pain in the region. Her dental history revealed that she had received five dental implants (August 2009). Three months after the procedure, she returned to her implantologist due to discomfort, swelling, and suppuration (October 2009). At the time, the dental implants placed in the region of Teeth #20 and #27 were then removed (Figure 1). A cone beam computed tomography (CBCT) scans taken after the surgical procedure (Figure 2) suggested that the dental implants in the region of Teeth #20 and #27 had been lost; there was also a hypodense area circumscribing the implant region at the site of Tooth #23, along with an osteolytic lesion and cortical destruction in the buccal region, the base of the mandible, the lingual region of the right anterior mandibular area, and the region of Tooth #27. Findings were suggestive of osteomyelitis (Figure 2). The patient reported that the implantologist prescribed oral antibiotics (not specified by the patient).

**Figure 3:**
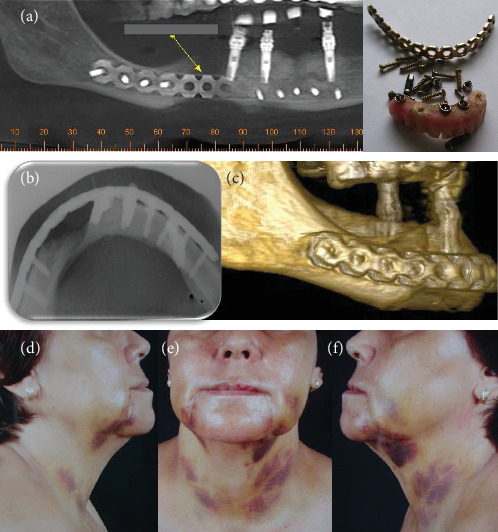
(a) CBCT scan shows the reconstructive plate associated with the radiolucent destruction area in the right quadrant of the mandible. (b) Reconstructive plate, implants, screws, and prosthesis removed from the mandible with infection. (c) Occlusal radiograph with the large radiolucent area. (d) 3D reconstruction of the right quadrant of the mandible. (e, f) Clinical appearance after hospital discharge.

In November 2009, the patient was referred to another specialist in oral and maxillofacial surgery for an evaluation of the possible risks of pathological fracture of the mandible. Because of the risk of mandibular fracture, the oral and maxillofacial surgeon placed a reconstructive titanium plate (2.4 mm), fixed with screws to the mandible (Figure 3). Ten days later (December 2009), the patient presented with facial and cervical swelling and a submandibular fistula with purulent discharge. The patient reported that she continued taking antibiotics, but there was no clinical improvement.

**Figure 4:**
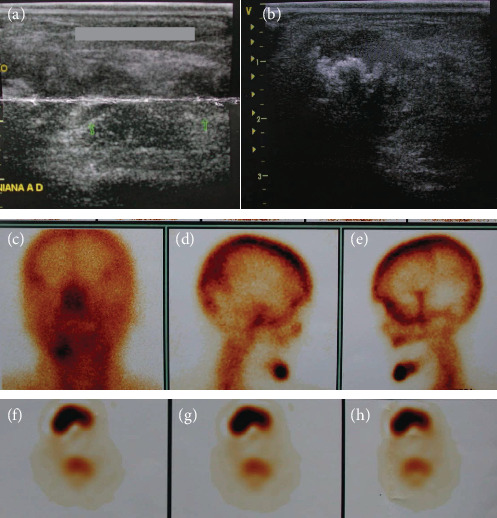
(a, b) Ultrasound image of the submental region with heterogeneous and hyperechoic areas, with acoustic shadowing and thickening compatible with swelling. (c–h) Scintigram shows marked uptake in the right side of the mandible.

**Figure 5:**
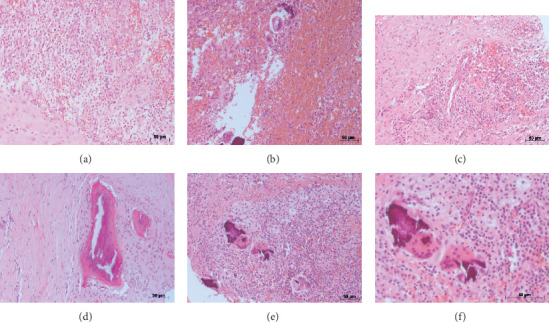
(a–e) Microscopic view of the material removed shows the area of chronic inflammatory infiltrate and devitalized bone surrounded by dense fibrous tissue heavily infiltrated by plasma cells, lymphocytes, and abscess (small mineralization areas) (hematoxylin–eosin; original magnification ×40). (f) ×100 magnification of the image shown in (e).

The distressed patient then sought care at the Neurologic Hospital of Goiânia and was referred to the oral and maxillofacial service, where the clinical signs and symptoms described above were confirmed. She also had a fever and was dehydrated. The patient was hospitalized immediately and stayed in the intensive care unit for 16 days. Several tests were requested: blood tests, culture, antimicrobial susceptibility tests, ultrasonography, scintigraphy, and computed tomography (CT). Blood tests revealed leukocytosis. The culture of oral secretions was positive for *Klebsiella pneumoniae* (Vitek, Biomerieux), and the microorganisms were resistant to ampicillin and amoxicillin. Imaging studies (CT, ultrasonography, and scintigraphy) revealed an osteolytic lesion and radiolucent area with cortical destruction in the buccal area and the base of the right quadrant of the mandible. The thickness and heterogeneity of the subcutaneous tissue in the submental region were consistent with edema. Surgery was planned for the removal of the reconstructive plate, two dental implants with peri-implantitis, and bone and necrotic tissue (particles of bone sequestrums), and the careful administration of systemic drugs was prescribed (Figures 3 and 4). The tissue sample (histopathology specimen) was sent to the clinical pathology service for microscopic examination, and the diagnosis of chronic suppurative osteomyelitis was confirmed. Microscopical findings revealed an area of chronic inflammation associated with an irregular fragment of necrotic bone surrounded by dense fibrous tissue heavily infiltrated by mononuclear plasma cells, lymphocytes, and neutrophilic granulocytes (Figure 5).

**Figure 6:**
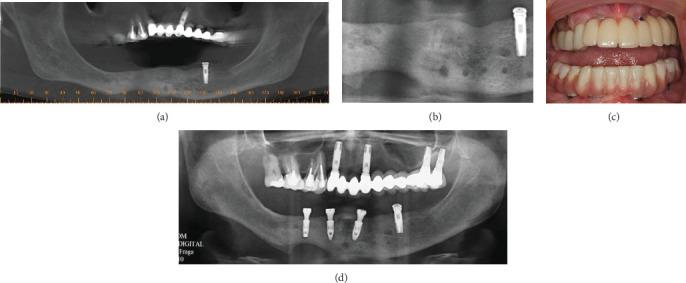
(a, b) CBCT scan shows bone remodeling in the area of osteomyelitis. (c, d) Implant follow-up and prosthetic rehabilitation 2 years later.

The patient received IV penicillin (4 million IU in 50 mL saline solution, four times a day for 15 days) and amikacin (1 g in 125 mL saline solution, once a day for 16 days). At hospital discharge, the patient was advised to continue taking 1 g cephalosporin three times a day for 15 days. Her clinical conditions returned to normal after 16 days. New dental implants were placed 12 months after the osteomyelitis treatment was completed (March 2011). Two years later in a follow-up visit, the patient was well, free of disease, and without any sign or symptom of infection in the mandible (April 2013). Prosthetic rehabilitation was carried out (Figure 6).

## 3. Discussion

Maxillofacial osteomyelitis is an inflammation of cortical and cancellous bone, which affects the mandible more frequently. Bacterial infections of different origin are important causes of osteomyelitis [1, 2, 6]. When chronic, osteomyelitis may be suppurative and produce an abscess, fistula, or bone sequestrum at some stage of the disease [3]. In the case described, mandibular osteomyelitis occurred secondary to the placement of five dental implants, a rare but serious complication. The patient presented with discomfort, swelling, suppuration, and a fistula, symptoms that contributed to a delayed diagnosis. This delay in removing the implants likely increased the risk of progression to severe osteomyelitis. A probable cause of implant failure was a bone infection, which ultimately led to the development of osteomyelitis [13].

Implant success, survival, and failure were discussed in a recent international congress of oral implantology, and Misch et al. [14] reported that a dental implant should primarily act as a support for prosthetic restorations, in the same way as a natural tooth root and crown. Success, difficult to describe for implants, ranges from health to disease in both conditions. The primary criteria for the assessment of implant quality or health are pain and mobility. The presence of either compromises implant quality, or removal is usually indicated. Implant failure, easier to describe than implant success or survival, may result from a variety of factors. The implant should be removed under any of the following conditions: (1) pain on palpation, percussion, or function; (2) horizontal and/or vertical mobility; (3) uncontrolled progressive bone loss; (4) uncontrolled exudate; or (5) more than 50% bone loss around the implant. Implants surgically placed but unable to be restored (sleepers) are also included in the failure group and are classified as failures in all statistical data, regardless of whether they remain in the mouth or are removed. Implants that have exfoliated or have been surgically removed are also included in the failure category.

The treatment of osteomyelitis is a long and complex process, often requiring prolonged antibiotic therapy and multiple surgical interventions [13, 15]. Conservative approaches are typically for patients in the chronic stage, making extensive segmental tissue removal a necessity in many cases [15]. While the decision to remove a recently placed implant can be challenging for both the clinician and the patient, it is often the most prudent course of action to prevent future complications. In the case presented, once osteomyelitis was diagnosed, the most rational approach involved the immediate removal of the reconstructive plate, the implants affected by peri-implantitis, and the necrotic tissue and bone sequestrums. This was combined with the careful administration of systemic medication to manage the infection. The criteria for implant failure were clearly identified, and the treatment strategy prioritized the immediate removal of the compromised implants to address the underlying pathology effectively.

Osteomyelitis may be secondary to a contiguous focus of infection (trauma, surgery, and infected joint prosthesis) to vascular insufficiency (diabetic foot infections) and to blood infections [5]. Trauma, bone surgery, and joint replacements, during which contamination may occur, may act as reservoirs of bacteria that reach the bone. Osteomyelitis associated with infected prostheses has an important clinical impact because they may often lead to extensive infection and difficult complications [5]. Montanaro et al. [16] reported that implant-related osteomyelitis is a severe and deep infection of bone that arises and develops all around an implant. *Staphylococcus aureus* contamination is the major cause of osteomyelitis, whether implant-related or not. Bone is an optimal substratum for *S. aureus* because these bacteria express various adhesins and adhere to bone proteins and to the biomaterial surfaces coated with proteins of the host's extracellular matrix. *S. aureus* not only colonize bone tissues but also invade and disrupt them by entering bone cells and inducing cell death and osteolysis. Osteomyelitis caused by *S. aureus* or enterobacteria is more common in other bones but may be found in the mandible less frequently. Chronic forms of osteomyelitis were investigated in bacteriological studies, but no bacteria were found in most samples [8].

Bacterial colonies attached to bone sequestrums in infected areas favor the organization of microorganisms on cortical and trabecular surfaces. Therefore, the susceptibility tests are not always accurate in chronic suppurative osteomyelitis, because several bacterial species may be in areas that cannot be reached using conventional means of collection, transportation, and cultivation. The detection of bacteria in tissue is not accurate when in colonies or biofilm [2]. Wu et al. [2] studied clinical and technical factors associated with culture results in histologically positive cases of osteomyelitis obtained from imaging-guided bone biopsies and found that the rate of positive culture results was low: Only 34% of the histologically positive cases had positive culture results. The aspiration of ≥ 2 mL of purulent fluid was associated with a significantly higher rate of positive cultures. Leonhardt, Renvert, and Dahlen [4] evaluated qualitative differences in the subgingival microbiota at the site of titanium implants and found clinical and radiographic signs of loss of supporting tissue (peri-implantitis) when compared with implants surrounded by health tissues. The microbiota of the healthy peri-implant sulci was like that found around teeth.

In the case described here, the culture of oral secretions was positive for *K. pneumoniae*. The susceptibility tests revealed that the microorganisms were resistant to ampicillin and amoxicillin. This case was difficult to treat, probably because the necrotic bone cavities protected the microorganisms from the action of antibiotics. For this reason, it was necessary to remove necrotic tissue and administer systemic drugs carefully to control infection.

The distinct imaging and clinical features of osteomyelitis indicate what steps are necessary to diagnose and treat this disease. Wu et al. [2] found a good correlation between both the histologic and microbiologic results of the imaging-guided biopsies and surgical procedures. Aside from one nondiagnostic surgical specimen, all surgical samples were positive for osteomyelitis and were histologically identical to the imaging-guided biopsy specimen. There were no differences between organisms isolated during imaging-guided biopsy and during surgery, which suggests that imaging-guided bone biopsies are accurate procedures.

In the case described here, imaging studies (CT, ultrasonography, and scintigraphy) showed a radiolucent area with cortical destruction of the buccal region and the base of the right quadrant of the mandible. Also, the thickness and heterogeneity of the subcutaneous tissue in the submental region were consistent with edema. Surgery was planned to remove the reconstructive plate, the implants with peri-implantitis, and bone and necrotic tissue, together with careful administration of systemic drugs.

In general, chronic inflammation, involving bone tissue with osteolysis, and the presence of abscesses and bone sequestrums are characteristics of a chronic suppurative osteomyelitis [5]. In our case, microscopical evaluation of the specimen removed showed an area of chronic inflammatory infiltrate associated with a nonvital bone surrounded by dense fibrous tissue heavily infiltrated by plasma cells, lymphocytes, and microabscesses with the presence of neutrophils, and these findings confirmed the diagnosis of chronic suppurative osteomyelitis.

Different treatment regimens have been suggested to treat osteomyelitis, such as rigorous antibiotic administration, removal of infected tissue, and adjuvant treatment with hyperbaric oxygen [6, 9, 10, 15, 17]. In the case described here, because of severe infection, the treatment prescribed was IV penicillin G and amikacin for 16 days while the patient was hospitalized and, after hospital discharge, 1 g cephalosporin three times a day for 15 days.

This case report underscores the potential for routine implant surgery to result in severe complications. Severe chronic suppurative osteomyelitis, despite the availability of modern antibiotics, continues to pose significant challenges in diagnosis and treatment. Patients should be informed about this rare but serious potential complication prior to undergoing implant procedures. Prompt recognition and immediate management of any infection following dental implant placement are essential to mitigate risks and prevent progression to severe outcomes.

## 4. Conclusion

The treatment of osteomyelitis should be carefully prescribed. Imaging studies, surgical removal of bone and necrotic tissue, and histopathological analyses are essential for the diagnosis.

## Data Availability

The data that support the findings of this study are available from the corresponding author upon reasonable request.
